# Inflammation and impaired endothelium-dependant vasodilatation in non obese women with gestational diabetes mellitus: preliminary results

**DOI:** 10.1186/1476-511X-12-93

**Published:** 2013-06-27

**Authors:** Ines Mrizak, Amel Arfa, Mariem Fekih, Haythem Debbabi, Ali Bouslema, Imen Boumaiza, Monia Zaouali, Naim A Khan, Zouhair Tabka

**Affiliations:** 1 Department of Physiology and Functional Exploration, Farhat Hached University Hospital, Sousse, Tunisia; 2 Department of Gynecology, Farhat Hached University Hospital Sousse, Sousse, Tunisia; 3 Department of Biochemistry, Sahloul University Hospital Sousse, Sousse, Tunisia; 4 INSERM U866, Physiology of Nutrition & Toxicology, University of Burgundy, Faculty of Life Sciences, Dijon, France

**Keywords:** Forearm skin blood flow, Laser Doppler flowmetry, Gestational diabetes mellitus, Inflammation

## Abstract

**Background:**

To evaluate whether abnormal endothelial function, a common finding in gestational diabetes mellitus (GDM) pregnancies, can be explained by inflammatory cytokines.

**Methods:**

Forearm skin blood flow (FSBF), into response to acetylcholine (Ach) (endothelium-dependent vasodilatation), were measured in 24 pregnant control subjects and 28 gestational diabetes mellitus (GDM) women, in the third trimester of gestation. A fasting glycemic and lipidic panel was obtained, and inflammatory cytokines (TNF-α and IL-6) and adiponectin were also determined.

**Results:**

FSBF is significantly reduced in GDM group compared with control subjects (344.59 ± 57.791 *vs*.176.38 ± 108.52, P < 0.05). Among all subjects, FSBF showed a strong negative correlation with TNF-α and IL-6 (r = −0.426, P < 0.0001 and r = −0.564, P < 0.0001, respectively) and positive correlation with adiponectin (r = 0.468, P < 0.0001).

**Conclusions:**

Endothelial function, an early marker of macrovascular disease, is present in non-obese pregnancies complicated by GDM. This alteration seems to be directly related to inflammatory status, which may represent a patho-physiological link between GDM and type 2 diabetes and, later on, metabolic syndrome.

## Introduction

Gestational diabetes mellitus (GDM), is one of the most commonly observed obstetrical complications affecting from 5% to 8% of all pregnancies [1,2].

GDM is associated with the development of inflammation [3], where adipose tissues play an important role in the regulation of insulin sensitivity by secreting adipokines which are involved in the pathogenesis pregnancy-induced insulin resistance, in the case obese pregnancies [4-6]. However, obesity may be not the only link between inflammation and glucose intolerance during pregnancy, placenta is also an important source of cytokines [7].

Many studies suggest that GDM is characterized by the installation of subclinical inflammation associated with a vascular dysfunction due to the insulin resistance, but to date there has been a dearth of studies, evaluating cytokine levels in women with GDM in order to explain their contribution to endothelium dysfunction [7]. Non-invasive measurement of microcirculatory blood flow in patients has recently emerged as useful tool to investigate the effect of insulin resistance on endothelial function [8,9].

We have conducted the present study to assess whether non-obese GDM women present an impairment of endothelial NO release; we wanted to focus us on the only effect of diabetes on the inflammatory status and its impact on endothelial function. Hence, we have investigated the endothelium-dependent vasodilatation by measuring non-invasively the change of FSBF in response to graded infusion of acetylcholine (Ach). We have also assessed whether the inflammatory cytokine levels can be involved in the impairment of endothelial function.

## Methods

### Subjects

The study was conducted in the Physiology and Functional Explorations Department, University Farhat Hached Hospital, Sousse, Tunisia, and it was approved by Farhat Hached Hospital Ethical Committee for research on humans in Tunisia. An informative written consent was approved and signed by all the women of the study.

A total of 28 GDM patients were selected between June 2010 to June 2011, from High Risk Pregnancy Department, Farhat Hached Maternity. Inclusion criteria were the following: normal glucose tolerance in early pregnancy, diagnosis of GDM in the 2^nd^ or the 3^rd^ trimester of gestation, according to the criteria of National Diabetes Data Group [10], the age range was between 20 and 39 years and gestational age was between 28^th^ and 41^th^ week of gestation.

The gestational age was derived from the last menstrual period, otherwise, gestational age was corrected on the basis of ultrasonographic measurements, consonant with clinical practice.

The selection of control individuals was from among pregnant women with the same characteristics including similar anthropometric parameters and gestational age, but with normal glucose tolerance. Hence, O’Sullivan test was conducted between the 24^th^ and 26^th^ week of gestation [11]. A total control subjects enrolled in the study was 24 pregnant women. Under the same conditions as the diabetic group we collected anthropometric measures of control group. The controls was measured in an earlier gestational week in order to eliminate any eventuality that controls may have glucose intolerance before it was based on monitoring records in High Risk Pregnancy Department, Farhat Hached Maternity. An O’Sullivan test was conducted between the 24^th^ and 26^th^ week of gestation.

Each of the 52 pregnant women who completed the study were first asked to provide details of medical and smoking history and then participated in health screening session and overall medical examination, including anthropometric measurement and resting arterial blood pressure.

In recruiting patients we held that the patients showed no risk to develop a preeclampsia, we followed the screening adopted by the High Risk Pregnancy Department, Farhat Hached Maternity, namely systolic blood pressure 120 mmHg and diastolic blood pressure 90 mmHg, and negative proteinuria because it will in the risk of influence endothelial function. And all GDM were monitored by insulin treatment.

All pregnancies were conducted under optimal conditions, all deliveries are vaginal, and took place between 36 and 41 weeks, and no special feature of fetal growth (data is not shown).

### Biochemical parameters

Blood samples were collected from subjects after 12 hours overnight fast, the blood was maintained at 4°C centrifuged, distributed in aliquots and stored at −80°C until the batched measurement of parameters. Serum total cholesterol (TC) and triglycerides (TG) were determined by standard assays. High-density lipoprotein cholesterol (HDL) was measured by direct assay. Low-density lipoprotein cholesterol (LDL) concentrations were calculated with the Friedwald formula [12]. Fasting glucose was measured by the glucose oxidase method. All biochemical parameters were determined on an automated Synchron CX7 Clinical System (Beckman, Fullerton, CA). Plasma insulin was assayed by IRMA Insulin kit (Immunotech, France). Insulin resistance (IR) was evaluated with the homeostasis model assessment (HOMA) using the following equation: HOMA-IR= (Fasting insulin (μUI/ml) * Fasting glucose (mmol/l) / 22.5 as per method of Matthews et al. [13].

Concentrations of maternal cytokines, including TNF-α and IL-6 were measured by ELISA-kits (Immunotech, France). Adiponectin concentrations were measured also by ELISA (R & D System, USA) according to the manufacturer’s instructions.

### Laser Doppler iontophoresis and hemodynamic measurement of FSBF

Combining Laser Doppler flow measurements with iontophoresis of vasoactive agents is a promising non-invasive tool and an attractive technique for studying the hemodynamic of the skin microcirculation in humans.

The protocol used is well-explained in the study of Miâdi-Messaoud et al. [8]. The equipments used were the following: Laser Doppler flowmeter (Periflux PF5000; Perimed, Stockholm, Sweden), electrode chamber (PF383; Primed Stockholm, Sweden) and acetylcholine chloride (Sigma Aldrich, Switzerland).

The following parameters were determined:

• Basal perfusion index (skin temperature = 33°C, arbitrary unit) during the second 2 min without Ach infusion

• Perfusion index after the third dose of Ach iontophoresis, i.e. the maximal endothelial response (arbitrary unit)

• Maximum perfusion index after heat hyperthermia and without Ach infusion (skin temperature = 44°C, arbitrary unit)

For all groups, cumulative concentration response curves (CCRC) of FSBF to Ach were obtained for each individual. For all groups, FSBF endothelium-dependant was defined as the maximal blood flow in response to Ach iontophoresis divided by the mean baseline blood flow expressed in percent.

### Statistical analysis

Statistical analyses are performed using SPSS 17.0. All variables measured are reported as mean (M) ± standard deviation (SD), by applying Kolmogrov-Smirnov normality test; we found that all variables are normally distributed so we applied parametric analyses using the student *t*-test. Spearman correlation was performed on all parameters with FSBF. All correlated variables are introduced in linear regression, for relationship between FSBF as dependant variable and the inflammatory cytokine as independent variables in ascendant model, to assess the magnitude of their individual effects on FSBF. A p-value < 0.05 was considered statistically significant for all tests.

## Results

Anthropometric parameters of the two groups are shown in Table 1. No significant differences in all anthropometric parameters between two groups were observed as regards age, weight, BMI, systolic blood pressure, diastolic blood pressure, waist circumference and hip circumference. In the two groups gestational age is homogenous, and there is no large variability.

**Table 1:** Anthropometric parameters

	**Control subjects**	**GDM**	**p**-**value**
Age (years)	28.3 ± 4.24	31.7 ± 5.5	0.064
Weight (kg)	76.1 ± 3.23	76 ± 2.3	0.239
Height (m)	1.6 ± 0.06	1.63 ± 0.04	0.200
BMI (kg/m^2^)	29.7 ± 2.23	28.5 ± 1.8	0.440
Systolic blood pressure (mmHg)	111.13 ± 5.6	113.01 ± 9.28	0.074
Diastolic blood pressure (mmHg)	61.46 ± 7.9	61.97± 10	0.298
Waist circumference (cm)	107.7 ± 11.66	115.5 ± 10.58	0.613
Hip circumference (cm)	109.7 ± 6.7	116.1 ± 11.92	0.036

Data are M ± SD. n=24 control subjects; n=28 GDM subjects.

Metabolic parameters are shown in Table 2. As expected, glucose at fasting, HOMA-IR and HbAc (glycated hemoglobin) were significantly higher in GDM group compared to control, whereas fasting insulinemia was insignificantly increased in GDM group.

**Table 2:** Comparison of metabolic and vascular characteristic between control subjects and GDM subjects

	**Control subjects**	**GDM**	**p-value**
Fasting glucose (mmol/l)	5.03 ± 0.46	5.42 ± 1.64*	0.01
Fasting insulinemia (μUI/ml)	10.59 ± 11.1	13.19 ± 10.05	0.67
Triglyceride (mmol/l)	1.99 ± 0.23	2.4 ± 0.93	0.38
Total cholesterol (mmol/l)	5.12 ± 1.26	5.43 ± 1.54**	<0.0001
HDL cholesterol (mmol/l)	1.47 ± 0.55	1.31 ± 0.3*	0.018
LDL cholesterol (mmol/l)	2.59 ± 1.39	2.94 ± 1.27	0.47
Apo A1 (g/l)	1.90 ± 0.7	1.50 ± 0.34**	0.001
Apo B (g/l)	1.36 ± 0.5	1.22 ± 0.43	0.64
Urea (mmol/l)	3.83 ± 1.16	2.44 ± 0.89*	0.050
Creatinine (μmol/l)	49.99 ± 0.15	50.74 ± 8.76	0.6
HbAc (%)	5.27 ± 0.5	6.7 ± 1.21*	0.002
HOMA-IR	1.53 ± 0.51	2.38 ± 1.39*	0.02
Adiponectin (μg/ml)	10.02 ± 1.1	8.58 ± 1.7*	0.01
IL-6 (pg/ml)	53.17 ± 1.45	77.84 ± 10.98*	0.002
TNF-α (pg/ml)	61.75 ± 6.5	71.63 ± 12.33	0.091
C-reactive protein (mg/dl)	0.86 ± 1.1	0.80 ± 1.4	0.716
FSBF endothelium-dependent (%)	1176.38 ± 531.67	344.59 ± 305.81*	0.037

Data are M ± SD. n=24 control subjects; n=28 GDM subjects and significant difference is as follow: *p<0.05, **p<0.001.

TC, creatinine were significantly higher in GDM group compared to control whereas triglyceride and LDL levels were higher in the same group but were not significantly different with the control group. The levels of HDL, Apo A1, adiponectin and urea were significantly decreased in GDM group compared to control.

FSBF was significantly decreased in GDM group compared with control. Significant correlations among all groups regarding FSBF with biochemical and inflammatory parameters are regrouped in Table 3. Positive correlation retained, with FSBF, was urea, ApoA1 and adiponectin. Negative correlation was obtained with creatinine, HbAc, HOMA-IR, IL-6 and TNF-α was retained with dependant variable, FSBF.

**Table 3:** **Spearman**’**s bivariate correlation coefficients FSBF with other variables**

	**r**	**p-value**
Urea	0.334	0.003
Creatinine	0.123	0.207
Apo A1	0.351	0.002
HbAc	−0.514	<0.0001
HOMA-IR	−0.334	0.003
Adiponectin	0.468	<0.0001
IL-6	−0.564	<0.0001
TNF-α	−0.426	<0.0001

In order to evaluate the independent contribution of biochemical and inflammatory parameters to predict FSBF, we performed an ascendant regression analysis only of IL-6, TNF-α and adiponectine in the model with a strong linear relationship with FSBF (r^2^ = 0.43, p < 0.0001), FSBF = 1738.57 – 0.372 * IL-6 – 0.249 * TNF-α + 0.246 * adiponectin.

## Discussion

The results of this study show the following new findings: 1) as compared with control pregnant women, non-obese GDM women show an impaired FSBF; and 2) FSBF is inversely correlated to pro-inflammatory cytokines, IL-6 and TNF-α, and in parallel related to adiponectin.

FSBF, an endothelium-dependant phenomenon, was examined at supine position. Ach induced vasodilatation of skin microcirculation by iontophoretic transdermal administration, involving agonistic endothelium-dependent response [14]. Ach provokes increases in the skin laser Doppler blood flow signal in a dose-dependent manner [15] and with an acceptable reproducibility (coefficients of variation between 10 and 17%) [16,17]. This technique was the method of choice for pregnant women in view of its non-invasiveness and the comfortable conditions of realization.

Although, many studies has explored the endothelium function with GDM women, but, to our knowledge, no previous study has assessed *in vivo* the maternal endothelial function in non-obese pregnancies, complicated by GDM, using combined Laser Doppler blood flow measurement with iontophoresis of vasoactive agent like Ach.

Our results are consistent with the evidence that women with pregnancies complicated by GDM have impaired endothelial function. This malfunction should be related to deregulation of glucose metabolism according to the literature [18]. There are several investigators who have also reported this impaired resistant in euglycemic women with previous GDM [18]. One of the principal objectives with regard to diabetic patients, at high-risk pregnancies, is to obtain an euglycemic level.

**Figure 1:**
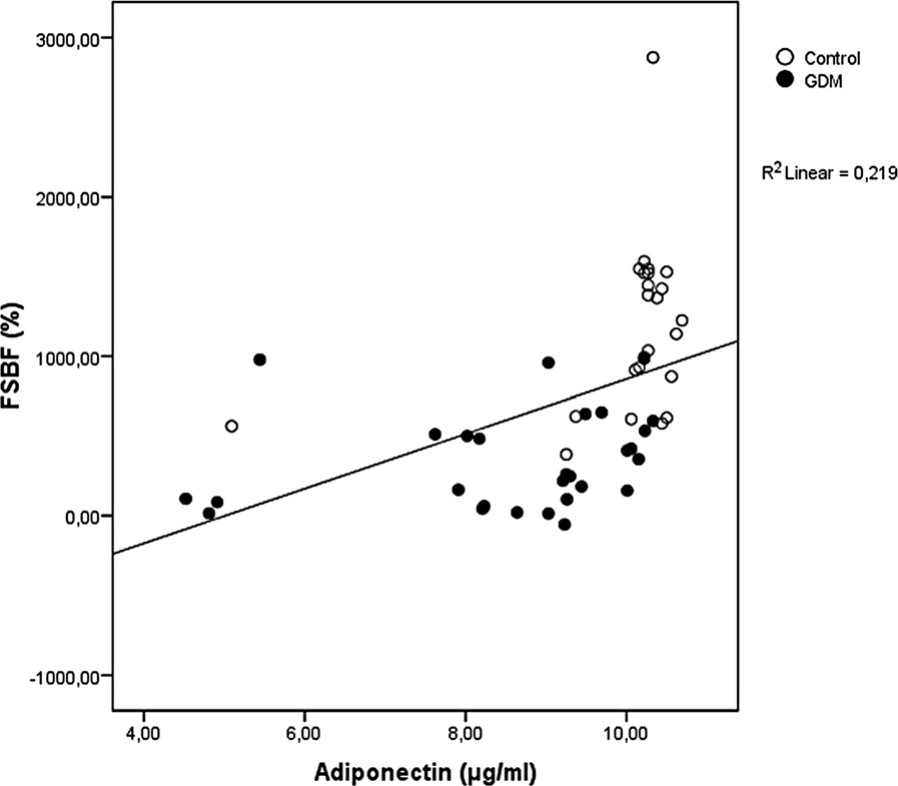
Linear regression analysis of percentage increase in FSBF and adiponectin.

**Figure 2:**
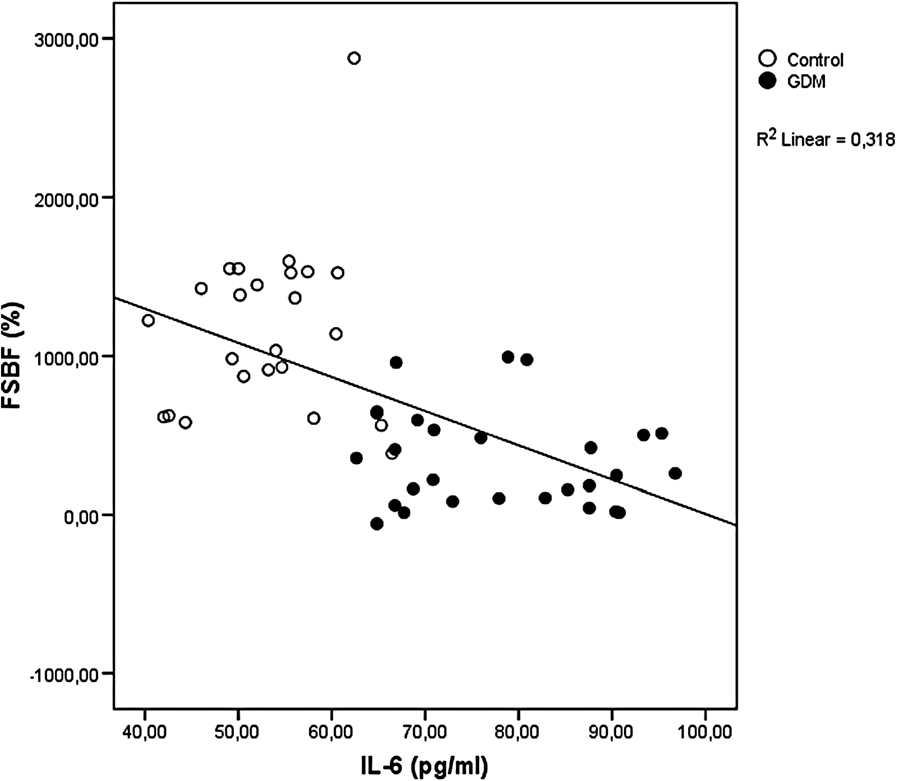
**Linear regression analysis of percentage increase in FSBF and IL-6.**

**Figure 3:**
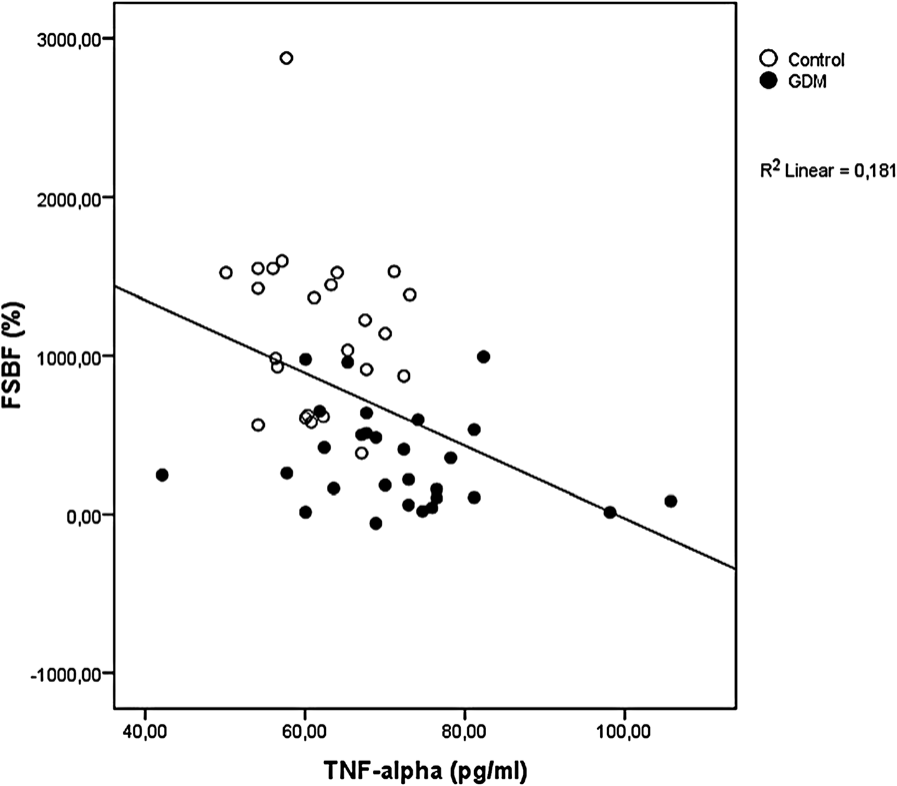
**Linear regression analysis of percentage increase in FSBF and TNF-α.**

Besides, endothelial function in diabetes has been attributed mainly to hyperglycemia [19,20] but also, and recently to subclinical inflammation [7]. It is interesting to note that among all subjects, FSBF is strongly and positively related to anti-inflammatory adiponectin (*r* = 0.468, P < 0.0001), as shown in Figure 1, and negatively related to the levels of pro-inflammatory cytokines, IL-6 and TNF-α (respectively, *r* = −0.564, p < 0.0001; *r*= − 0.426, p < 0.0001), as shown respectively in Figures 2 and 3.

In our study, insulin resistance, performed with HOMA system, was significantly higher in GDM group compared with control, and it was negatively correlated with FSBF. It has been reported that there is a relationship between insulin resistance and inflammatory status as described by Dandona et al. [21].

According to our results, it seems that the GDM patients might suffer from metabolic syndrome, marked with elevated triglyceride and LDL concentration, low HDL concentrations. This physiological change might be the result of an elevated triglyceride load in the HDL particles by the action of hepatic lipase which hydrolyses the triglycerides [21]. The small HDL particles, which have lost triglyceride, are filtered by the kidney, resulting in a decrease in apolipoprotein (Apo) A and HDL concentrations. Moreover, there are reports that insulin may promote ApoA gene transcription [22,23]. Hence, insulin resistance may be related to diminished ApoA biosynthesis, which seems to be the case in our GDM subjects [24].

In the light of our results, we can propose that there exists a tridimensional relationship between FSBF, insulin resistance and inflammatory status in GDM group [21,25]. However, the relationship between pro-inflammation and endothelial function is not well-understood. In human endothelial cells, L-arginine is taken up via membrane transport systems grouped a family of proteins known as cationic amino acid transporters (CATs) (hereafter referred as “CATs family”) [26] whose expression and activity, and the mechanisms modulating these phenomena, have been extensively described [27-32], including in the human placenta [33,34].

In another hand, it is now established that GDM are pathological conditions altering hCAT (cationic amino acid transporter-1)-mediated arginine transport and eNOS (endothelial nitric oxide synthase) synthesis of NO in human feto-placental vasculature, due to abnormal signaling pathway leading to altered vascular reactivity and changes in umbilical vessels blood flow from and to fetus with serious consequence on its growth [35]. Altogether these findings could be crucial for fetal insulin modulation of endothelial-derived NO synthesis in human umbilical vessels from pregnancy diseases associated with hyperinsulinemia, such as GDM, and other states of insulin resistance [27,36-39].

It has been reported that Akt2-null mice develop insulin resistance and mild hyperglycemia with hyperinsulinemia [40] as Akt2 is a key protein involved in signal transduction. Besides, the phosphorylation of Akt2 can be induced by TNF-α and IL-6 which have recently been implicated in inducing SOCS-3 [41-43], a protein known to interfere with tyrosine phosphorylation of the insulin receptor and IRS-1 (insulin receptor substrate 1) [44]. This, in turn, reduces the activation of Akt (protein kinase B), which normally causes the translocation of the insulin-responsive glucose transporter, Glut-4, to the plasma membrane. It also induces the phosphorylation of the enzyme NOS and its activation to generate NO [42].

As discussed above, NO is a key mediator of vascular health, promoting smooth muscle relaxation and exerting anti-inflammatory and antithrombotic effects. Under normal conditions, eNOS transfers electrons to L-arginine to produce citrulline and NO. Decreased NO availability has been observed in clinical studies of patients with insulin resistance [45,46].

What is the origin of this pro-inflammatory state in the GDM women? It is possible that during insulin resistance, increased IL-6 not only diminishes insulin sensitivity but by suppressing insulin signal transduction also interferes with anti-inflammatory effect of insulin, and might favour inflammation during insulin resistance [47].

Indeed, novel non-metabolic actions have been attributed to insulin, like anti-inflammatory effect [21]. Insulin has been shown to suppress several pro-inflammatory transcription factors, such as nuclear factor (NF-κB), Egr-1 (early growth response protein 1) and activating protein-1 (AP-1) and the corresponding genes which mediate inflammation [47,48].

The pro-inflammatory state induces insulin resistance, leading to clinical and biochemical manifestations of the metabolic syndrome. This resistance to insulin action promotes inflammation further through an increase in FFA (Free fatty acid) concentration and interferes with the anti-inflammatory effect of insulin [21]. The increase of FFA may be the result of hormonal modifications occurs until gestation like Human placental lactogen (HPL).

Insulin secretion in women with GDM is defective and, therefore, is unable to rise adequately to compensate for the insulin resistance; the result is hyperglycemia. The mechanism by which elevated plasma FFA levels cause insulin resistance in skeletal muscle includes intramyocellular accumulation of diacylglycerol, which activates protein kinase C (the b II and d isoforms). This results in reduction of tyrosine phosphorylation of the insulin receptor substrate-1 and inhibits activation of phosphoinositol-3 kinase, an enzyme that is essential for normal insulin-stimulated glucose uptake [49,50].

Inflammatory cytokines have a pivotal role in placental function throughout pregnancy. Moreover, the progressive development of insulin resistance during pregnancy is due, in part, to placental cytokines such as TNF-α [51] and leptin [52]. Studies on the expression of inflammatory genes in GDM women have demonstrated major changes in placental gene expression [53].

## Conclusion

The particularity of our study is that the studied population was not obese; however, the results showed clearly that the GDM group suffered from dyslipidemia, insulin resistance, pro-inflammatory status and exhibited endothelial dysfunction. Our study is limited by the number of subjects and these results should be supported by researches that extend the number of recruited.

## Abbreviations

Akt: Protein kinase B; Apo A1: Lipoprotein 1; Apo B: Lipoprotein B; ELISA: Enzyme linked immunosorbent assay; eNOS: Endothelial nitric oxide synthase; Erg1: Early growth response protein 1; FFA: Free fatty acid; FSBF: Forearm skin blood flow; GDM: Gestational diabetes mellitus; HbAc: Glycated hemoglobin; h-CAT: Cationic amino acid transporter 1; HOMA-IR: Homeostasis model assessment-insulin resistance; IL-6: Interleukin 6; IRMA: Immunoradiometric assay; IRS-1: Insulin receptor substrate 1; NF-κB: Nuclear factor-kappa B; NO: Nitric oxide; SCOS-3: Suppressor of cytokine signaling 3; T2D: Type 2 diabetes; TC: Total cholesterol; TG: Triglyceride; TNF-α: Tumor necrosis factor alpha.

## Competing interests

The authors declare that they have no competing interests.

## Authors’ contributions

MI was in charge of the practical work and prepared major parts of the manuscript. AA collected and analyzed data with the involvement of MI. FM participated in interpretation of the gynecology function. DH interpreted Laser Doppler Data. BA and BI conducted biochemical analyses. MZ conducted hormonal analyses. TZ and KNA planned and organized the study and contributed to the revisions and the final drafts of the manuscripts. All authors read and approved the final manuscript.
